# Dose differences between patients treated with MR-only, CT-only, or MR-CT fusion radiotherapy for prostate cancer

**DOI:** 10.1093/bjr/tqaf159

**Published:** 2025-07-16

**Authors:** Jonathan J Wyatt, Stephen Hedley, Neil Richmond, Serena West, Rachel L Brooks-Pearson, Tracy Wintle, Rachel A Pearson

**Affiliations:** Northern Centre for Cancer Care, Newcastle upon Tyne Hospitals NHS Foundation Trust, Newcastle, NE7 7DN, United Kingdom; Translational and Clinical Research Institute, Newcastle University, Newcastle, NE4 5PL, United Kingdom; Northern Centre for Cancer Care, Newcastle upon Tyne Hospitals NHS Foundation Trust, Newcastle, NE7 7DN, United Kingdom; Northern Centre for Cancer Care, Newcastle upon Tyne Hospitals NHS Foundation Trust, Newcastle, NE7 7DN, United Kingdom; Northern Centre for Cancer Care, Newcastle upon Tyne Hospitals NHS Foundation Trust, Newcastle, NE7 7DN, United Kingdom; Northern Centre for Cancer Care, Newcastle upon Tyne Hospitals NHS Foundation Trust, Newcastle, NE7 7DN, United Kingdom; Translational and Clinical Research Institute, Newcastle University, Newcastle, NE4 5PL, United Kingdom; Northern Centre for Cancer Care, Newcastle upon Tyne Hospitals NHS Foundation Trust, Newcastle, NE7 7DN, United Kingdom; Northern Centre for Cancer Care, Newcastle upon Tyne Hospitals NHS Foundation Trust, Newcastle, NE7 7DN, United Kingdom; Translational and Clinical Research Institute, Newcastle University, Newcastle, NE4 5PL, United Kingdom

**Keywords:** MR-only radiotherapy, MR, synthetic CT, prostate cancer

## Abstract

**Objectives:**

Magnetic Resonance (MR)-only radiotherapy has been clinically implemented but its dose impact has not been assessed in clinical practice. This study evaluated the volume and dose differences between patients treated with CT-only, MR-CT fusion, or MR-only prostate radiotherapy pathways.

**Methods:**

Four hundred fifty-four patients from a single centre were treated using MR-only (*n* = 96), CT-only (*n* = 248), or MR-CT (*n* = 110) pathways. Patients were selected for different pathways based solely on geographic location and treatment date. Patients were contoured by the same group of delineators and were planned for 60 Gy in 20 fractions by an automated planning algorithm. Clinical Target Volume (CTV), bladder, penile bulb and rectum volumes, and clinical dose constraints were compared with Kurshkal-Wallis tests, adjusted for multiple testing with a P<.05 significance level.

**Results:**

Median MR-only CTVs were $5\;{\text{cm}}^{3}$ smaller than CT-only (P=.004). Penile bulb Dmean was 12.0 Gy (CT-only), 9.1 Gy (MR-CT), and 5.9 Gy (MR-only, P<.001), with 79.0%, 90.9%, and 95.8% of patients within constraint. Rectum D$2\;{\text{cm}}^{3}$ was 57.4 Gy (CT-only), 57.6 Gy (MR-CT), and 56.5 Gy (MR-only, P<.001), with 35.1%, 20.9%, and 56.2% of patients within rectum V60 Gy constraint.

**Conclusions:**

The MR-only pathway produced significant reductions of 13% in CTV volume, 51% in penile bulb Dmean, and 2% in rectum D$2\;{\text{cm}}^{3}$ compared to CT-only.

**Advances in knowledge:**

The dose benefit from MR-only has been assessed in clinical practice, demonstrating significant reductions in penile bulb and rectum doses compared to both CT-only and MR-CT pathways. This suggests the MR-only pathway is required to provide the full benefit of MR contouring to reduce toxicities from prostate radiotherapy.

## Introduction

Magnetic Resonance (MR)-only radiotherapy with commercially available synthetic Computed Tomography (sCT) algorithms is becoming an established treatment pathway for prostate cancer, with multiple clinical implementations reported in the literature.^1–3^ The primary rationale given for MR-only radiotherapy is the ability to use the superior soft-tissue contrast of MR for delineation without the uncertainty of the MR-CT registration,^4^ with additional benefits such as reduction in concomitant dose due to MR using nonionizing radiation and improvements in departmental efficiency.^5^ The improved delineation of MR may lead to reductions in prostate target volume.^6^^,^^7^ Several studies have retrospectively reported average per-patient reductions in prostate target volume between MR-only and MR-CT pathways of 18% in 10 patients^8^ and 8% in 20 patients.^3^ These results were surprising since they were comparing delineations done on the same MR image in both cases. They, thus, show the impact of the MR-CT registration in the delineation process and suggest that MR-only radiotherapy may potentially result in reduced Organ At Risk (OAR) doses even without reductions in Planning Target Volume (PTV) margins.^8^

The next step would be to evaluate the volume and dose differences in CT-only, MR-CT, and MR-only prostate radiotherapy plans as they are used in clinical practice. The ability to assess small systematic differences between the different pathways depends on ensuring there are no other confounding systematic differences between pathways and minimizing the inter-patient variation within and between pathways. Although the biggest causes of inter-patient variation are due to patient factors such as size of OARs, other significant factors would include inter-observer variability in delineations and treatment plans.

Our centre has treated prostate radiotherapy patients using CT-only, MR-CT fusion, and MR-only pathways during the last 2 years, with patients assigned different pathways solely due to when and at which geographical site they were treated. Patients were all delineated by the same group of clinicians and dose planners using the same contouring guidelines, irrespective of which pathway or geographic site they were treated on. This would ensure there were no systematic differences between pathways in target and OAR delineations. In addition, patients were all planned using a fully automated treatment planning solution for prostate radiotherapy.^9^ This would not only ensure no systematic differences between pathways in treatment plans, but would also remove inter-planner variability, reducing the overall inter-patient variation. Therefore, the aim of this study was to evaluate the volume and dose differences between patients treated with CT-only, MR-CT fusion, or MR-only radiotherapy all planned with the same fully automatic algorithm.

## Methods

### Patients and treatment characteristics

Four hundred fifty-four prostate radiotherapy patients treated at Northern Centre for Cancer Care, Newcastle, UK between May 2023 and December 2024 were included in this retrospective study. Patients consent for their data to be used for audit/research purposes in the consent for treatment. Patients with hip prostheses or external contours larger than the MR scanner field of view (50 cm) were excluded. Patients were treated using either a MR-only pathway (*n* = 96), a CT-only pathway (*n* = 248) or a MR-CT fusion pathway (*n* = 110). Patients were selected for different pathways depending on geographic location and date of treatment (the main site was CT-only from May 2023 until June 2024 due to capacity issues and MR-only from June 2024 onwards, the satellite site was MR-CT fusion throughout). The patients ages were very similar between the different pathways, with median ages (interquartile range) of 73 years (70-77 years), 74 years (68-77), and 75 years (70-77) for CT-only, MR-CT, and MR-only pathways respectively. All patients were being treated for node negative intermediate to high risk prostate cancer.

**Figure 1.**
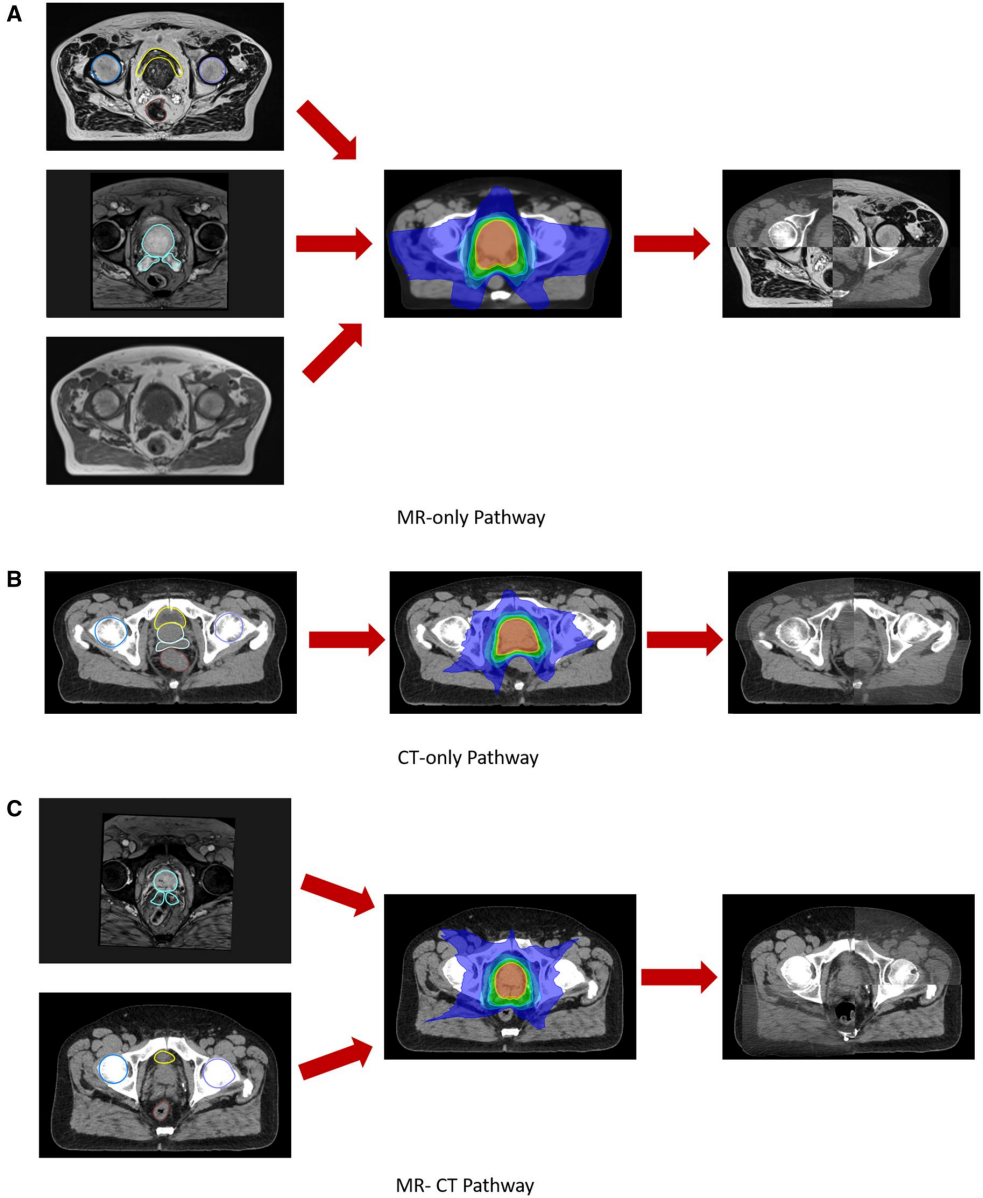
Diagrams of the three different pathways patients were treated with. (A) MR-only pathway showing on the left large field of view MR for OAR delineation (top, bladder in yellow, femoral heads in blue and purple, rectum in brown), small field of view MR for prostate and seminal vesicles delineation (middle image, light blue contour), and Dixon MR for sCT generation (bottom). In the middle is the dose distribution on the sCT and on the right is the CBCT registered to the large field of view MR. (B) CT-only pathway showing on the left the CT with OAR and target contours (same colours as in (A), in the middle dose distribution on the CT and on the right CBCT registered to CT. (C) MR-CT pathway showing on the left small field of view MR for target delineation (top) and CT for OAR delineation (bottom). In the middle is the dose distribution on CT and on the right CBCT registered to CT.

In the MR-only pathway, patients received a planning MR scan (Magnetom Sola, Siemens, Erlangen, Germany) on a flat couch-top (Civco Medical Solutions, Coralville, Iowa, USA) with a coil bridge for the anterior MR coil using external lasers for patient alignment. Patients were scanned with three sequences: T2-weighted, T2*-weighted and Dixon. The T2-weighted image was a large field of view 3D turbo spin echo sequence and was used for OAR contouring and on-treatment image matching to CBCT. The T2*-weighted image was a small field of view 2D multiple echo sequence used for target contouring. The Dixon image was a large field of view 3D Dixon sequence and was used for synthetic (s)CT generation. The sCT was produced with the Deep Learning continuous Hounsfield Unit algorithm on the scanner (Siemens) and used for plan optimization and dose calculation (Figure 1).

In the CT-only pathway, patients received a planning CT scan (Confidence, Siemens) on a flat couch-top using external lasers for patient alignment. Patients were scanned with a tube voltage of 120 kVp, a slice thickness of 3 mm and an effective tube current-exposure time of 88 mAs. The CT was used for target and OAR contouring, plan optimization and dose calculation, and on-treatment image matching to CBCT (Figure 1).

In the MR-CT fusion pathway, patients received both planning MR (Magnetom Sola, Siemens) and CT scans (Go Open Pro, Siemens) on flat couch tops using external lasers for patient alignment. The MR sequence consisted of just the small field of view T2*-weighted 2D multiple echo sequence for target contouring, with the CT being used for OAR contouring, plan optimization and dose calculation, and on-treatment image matching to CBCT (Figure 1).

In all three pathways patients were scanned using a combined customisable foot and knee rest (Civco Medical Solutions). Patients were imaged following routine bladder preparation consisting of an empty bladder 30 min prior to the scan, followed by drinking 400 mL of water, and bowel preparation consisting of the application of a micro-enema 60 min prior to the scan followed by bowel emptying.

All patients from both sites were contoured in RayStation (v9B and v2023B, RaySearch Laboratories, Stockholm, Sweden) according to the same clinical guidelines by the same group of seven clinicians and eight dose planners. The prostate CTV was anatomically defined as the prostate plus proximal centre of seminal vesicles 1 cm above the origin and any extra prostatic extension and the prostate and seminal vesicles CTV as prostate plus entire seminal vesicles.^10^ The same PTV margins were applied: 4 mm to prostate CTV and 8 mm to prostate and seminal vesicles CTV.^10^ All patients were prescribed 60 Gy in 20 fractions to the prostate PTV and 47 Gy in 20 fractions to the prostate and seminal vesicles PTV.^10^ All treatment plans used a single $360^{o}$ arc VMAT plan in RayStation and were created using the same fully automated planning algorithm. This was an in-house developed script which uses dynamic objectives and Pareto navigation techniques to generate a plan for a specific patient which balance trade-offs in PTV coverage and OAR doses that are consistent with trade-offs chosen by clinicians during a prior calibration of the system. The script was based on methods described previously.^9^^,^^11^^,^^12^ All patients were treated on TrueBeam or TrueBeam STx linear accelerators (v2.7, Varian Medical Systems, Palo Alto, USA) with daily online image matching using CBCT.

### MR-only dose calculation quality assurance

For patients in the MR-only pathway only, the dose calculation accuracy of the sCT was quality assured by recalculating the plan on the first fraction CBCT. Doses were calculated using bulk density over-rides with patient-specific thresholds in RayStation.^13^ The CBCT image was automatically converted into six tissue classes: air - $0.00121\;g{\text{cm}}^{-3}$, lung - $0.26\;g{\text{cm}}^{-3}$, adipose - $0.95\;g{\text{cm}}^{-3}$, tissue - $1.05\;g{\text{cm}}^{-3}$, cartilage/bone - $1.6\;g{\text{cm}}^{-3}$, and other - $3.0\;g{\text{cm}}^{-3}$. The difference in dose to the prostate PTV D50 between first fraction CBCT and sCT (CBCT—sCT) was calculated. Patients were assessed compared to previously published tolerances of [−2%,1%].^13^ Differences outside of these tolerances were investigated further.

### Comparison of volumes and planned doses

Patient treatment plans from each pathway were compared using ProKnow (v2.0.2.0, Elekta Solutions AB, Stockholm, Sweden). Contoured volumes of prostate-only CTVp, prostate and seminal vesicles CTVpsv, bladder, rectum, and penile bulb were compared. Doses to PTV D2, D50 and D98 were assessed to ensure there were no systematic differences in PTV doses between the pathways. Doses to OARs were selected based on published dose-effect relationships. For the bladder, long-term grade ≥2 toxicity has a dose-effect relationship with the D2cc.^14^ The penile bulb mean dose has shown to be predictive of erectile dysfunction, with a dose constraint of ≤20 Gy.^15^ For the rectum, long-term grade ≥2 toxicity has a dose-effect relationship with the D2cc and D50%.^16^ In addition, rectum constraints derived from clinician reported outcomes in the CHHiP trial were included, V60 Gy≤0.01% and V50 Gy≤22%.^17^

Each parameter was tested for at least one pathway being statistically significantly different to the others using a Krushkal-Wallis test. A significance level of P<.05 was used with *P*-values adjusted for multiple testing using the Benjaminini-Yekutieli method for false discovery control when tests are not independent.^18^ This ensured that false positive rate was maintained at 0.05. All statistics were calculated using scipy (version 1.13.1).

## Results

### MR-only dose calculation quality assurance

**Figure 2.**
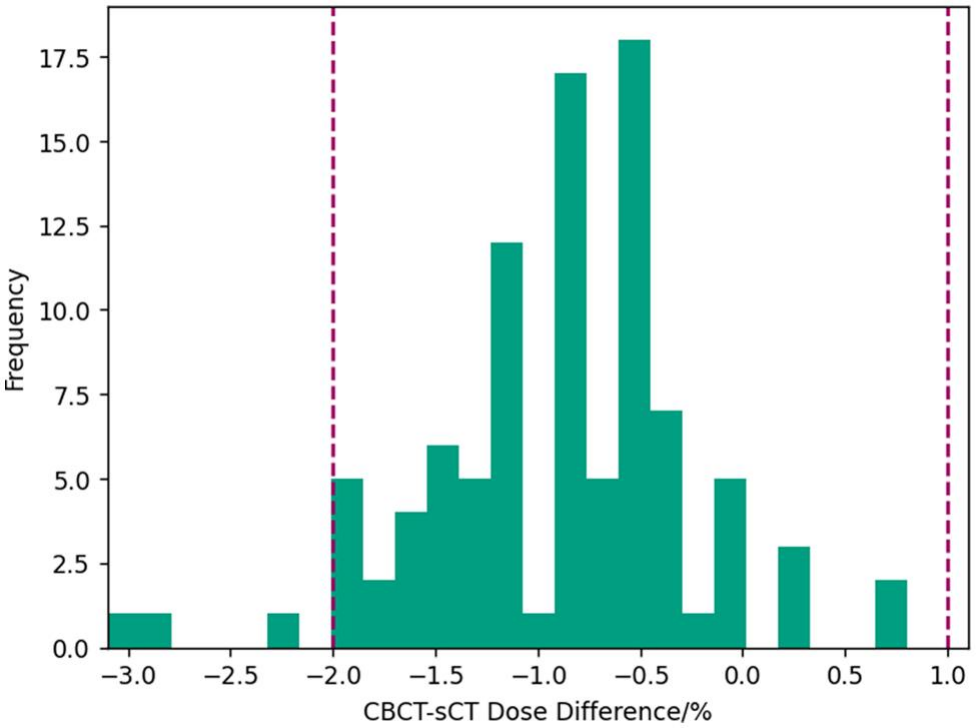
Histogram of dose differences in the primary PTV D50% between sCT and first fraction CBCT (green bars). Purple dashed lines indicate [−2%,1%] tolerances.

The mean dose difference to the primary PTV D50% was −0.89±0.07% (± standard error, minimum −3.1%, maximum 0.8%). Ninety-three out of ninety-six patients were within the previously determined tolerances of [−2%,1%] (Figure 2).^13^ Those patients who were outside had clearly gained weight in between planning MR image and the first treatment fraction.

### Comparison of volumes and planned doses

**Figure 3.**
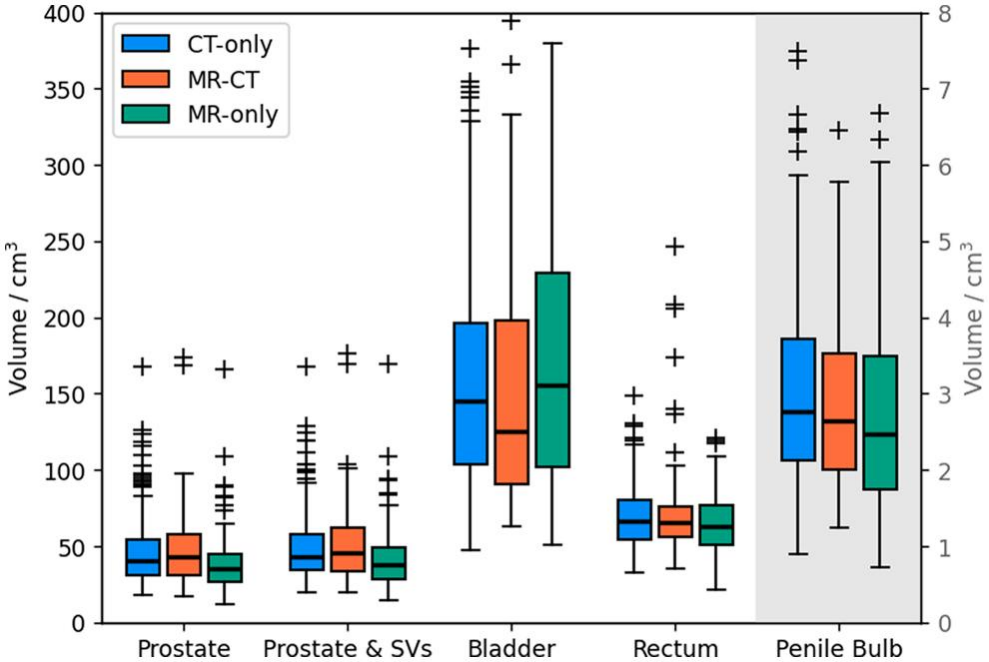
Boxplot of organ volumes for the CT-only (green), MR-CT (blue), and MR-only (orange) pathways. The penile bulb volumes are given on the second *y*-axis as they are so much smaller than the other organs. The horizontal lines indicate the median, the boxes the interquartile range, the whiskers the farthest point within 1.5× the interquartile range from the box and the crosses all outlier points.

The median CTVp volumes were statistically significantly different for at least one pathway (P=.004), with median values of $40_{-9}^{+14}\;{\text{cm}}^{3}$ ($\begin{array}{c} + \end{array}$ value to third quartile, − value to first quartile), $43_{-12}^{+15}\;{\text{cm}}^{3}$ and $35_{-8}^{+10}\;{\text{cm}}^{3}$ for the CT-only, MR-CT, and MR-only pathways respectively (see Figure 3). The median CTVpsv volumes were also statistically different (P=.004) with volumes of $43_{-9}^{+15}\;{\text{cm}}^{3}$ (CT-only), $46_{-12}^{+16}\;{\text{cm}}^{3}$ (MR-CT), and $38_{-10}^{+11}\;{\text{cm}}^{3}$ (MR-only). There was no statistically significant difference in OAR volumes (bladder P=.54, penile bulb P=.41, and rectum P=1.0).

**Figure 4.**
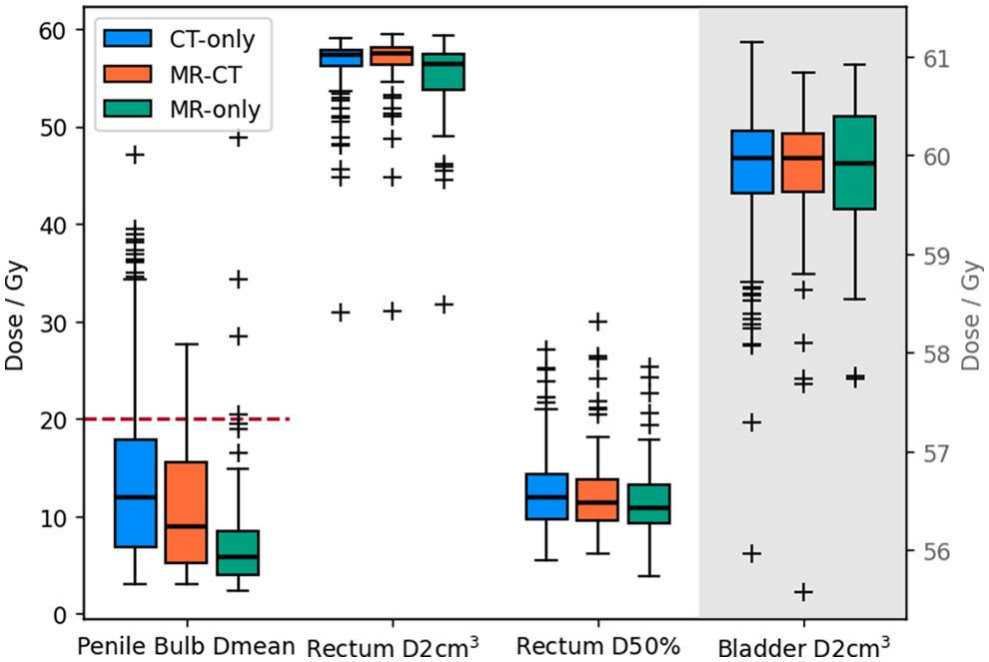
Boxplot of doses at different dose constraints for the CT-only (green), MR-CT (blue), and MR-only (orange) pathways. The purple dashed line indicates the tolerance for the relevant dose constraints where they exist. The bladder D2 cm^3^ are given on the second *y*-axis. The horizontal lines indicate the median, the boxes the interquartile range, the whiskers the farthest point within 1.5× the interquartile range from the box and the crosses all outlier points.

**Figure 5.**
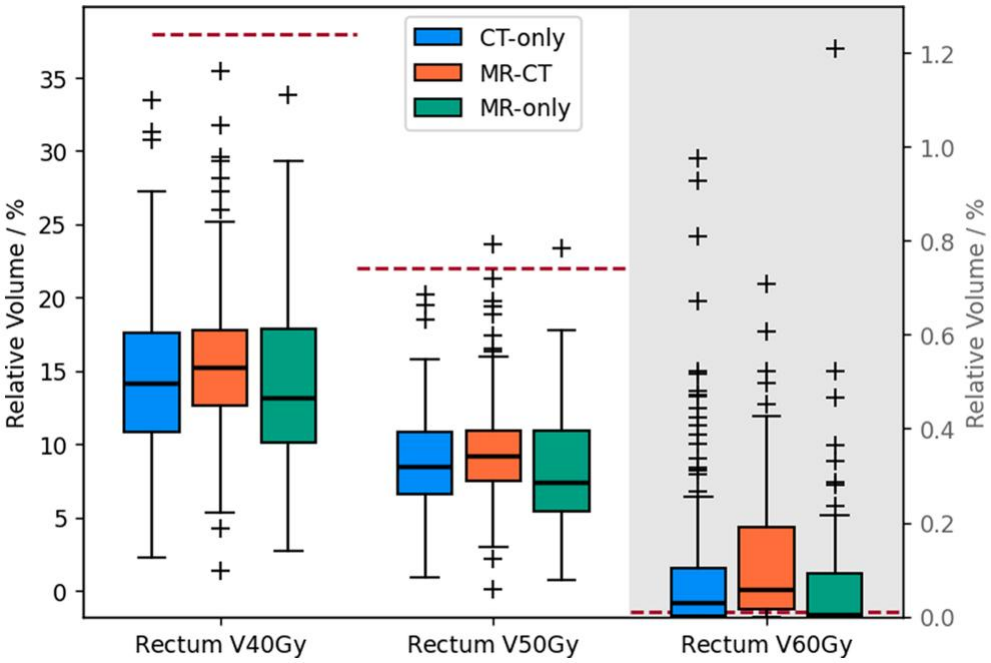
Boxplot of relative volumes at different dose constraints for the CT-only (green), MR-CT (blue), and MR-only (orange) pathways. The purple dashed line indicates the tolerance for the relevant constraints where they exist. The V60 Gy is plotted on a second *y*-axis since these were much smaller than for the other relative volume constraints. The horizontal lines indicate the median, the boxes the interquartile range, the whiskers the farthest point within 1.5× the interquartile range from the box and the crosses all outlier points.

The median doses to the PTV D2%, D50%, and D98% were within 0.1 Gy of each other for each pathway. Similarly the interquartile ranges of doses to the PTV D2% and D50% were within 0.1 Gy of each other, and to the PTV D98% within 0.2 Gy. There were statistically significant differences between at least one pathway for the mean dose to the penile bulb and the high doses to the rectum (D$2\;{\text{cm}}^{3}$ and V60 Gy), with the MR-only pathway having the lowest values (see Figures 4 and 5). The bladder D$2\;{\text{cm}}^{3}$ and the lower doses to the rectum (D50%, V50 Gy, and V40 Gy) were not significantly different. The MR-only pathway had higher proportions of patients within dose constraints for the penile bulb and the rectum V60 Gy (Table 1) than CT-only or MR-CT. The CT-only pathway had slightly higher rates of patients within constraints for the rectum V50 Gy despite the MR-only having the lowest median value. This was due to one outlier MR-only patient with a relative volume above the constraint (Figure 5).

**Table 1.** Doses and percentage of patients within different OAR constraints for each pathway.^a^

Organ	Constraint	CT-only		MR-CT		MR-only		*P*-value
		*Median*	*Within/*%	*Median*	*Within/*%	*Median*	*Within/*%	
Bladder	D$2{\text{cm}}^{3}$ [Gy]	$60.0_{-0.4}^{+0.3}$	–	$60.0_{-0.4}^{+0.2}$	–	$59.9_{-0.5}^{+0.5}$	–	1.0
Penile Bulb	Dmean [Gy]	$12.0_{-5.1}^{+5.9}$	79.0	$9.1_{-3.9}^{+6.5}$	90.9	$5.9_{-1.9}^{+2.7}$	95.8	<.001
Rectum	D$2{\text{cm}}^{3}$ [Gy]	$57.4_{-1.3}^{+0.5}$	–	$57.6_{-1.2}^{+0.6}$	–	$56.5_{-2.8}^{+1.0}$	–	<.001
	D50% [Gy]	$12.0_{-2.4}^{+2.3}$	–	$11.5_{-1.9}^{+2.3}$	–	$10.9_{-1.6}^{+2.4}$	–	.42
	V60Gy [%]	$0.0_{-0.0}^{+0.1}$	35.1	$0.1_{-0.0}^{+0.1}$	20.9	$0.0_{-0.0}^{+0.1}$	56.2	<.001
	V50Gy [%]	$8.5_{-1.9}^{+2.4}$	100.0	$9.2_{-1.7}^{+1.7}$	99.1	$7.4_{-2.0}^{+3.5}$	99.0	.12
	V40Gy [%]	$14.1_{-3.3}^{+3.5}$	100.0	$15.3_{-2.7}^{+2.5}$	100.0	$13.1_{-3.0}^{+4.7}$	100.0	.541

a The *P*-value shown is the result of Krushkal-Wallis test showing the significance of the differences between at least one pathways with the others. The units of each row are given in the square brackets in the constraint column.

## Discussion

This study has evaluated volume and dose differences in clinical treatment plans between MR-only, MR-CT, and CT-only pathways when delineated by the same cohort of clinicians and dose planners and planned using the same fully automatic planning algorithm. Prostate and seminal vesicles CTV volumes were statistically significantly smaller for MR-only patients by approximately $5\;{\text{cm}}^{3}$. OAR volumes were not significantly different. The MR-only pathway had statistically significantly lower mean doses to the penile bulb and the rectum D$2\;{\text{cm}}^{3}$ and V60 Gy, with higher proportions of patients within dose constraints for these parameters. There were no significant differences in doses to PTV, bladder or rectum D50%, V50 Gy, and V40 Gy.

The median prostate CTV and prostate and seminal vesicles CTV volumes were $5\;{\text{cm}}^{3}$ lower for the MR-only pathway, a difference which was statistically significant (P=.004 for both) despite the large variation between patients. It is widely reported that MR-defined prostate volumes are smaller than CT-defined volumes,^6^^,^^19^^,^^20^ which fits well with the data reported here. Interestingly, MR-CT CTV volumes had a similar distribution to CT-only (Figure 4) and in fact had larger median volumes. This is potentially due to clinicians adjusting CTV following registration with CT to account for small MR-CT registration inaccuracies.^3^ This fits with studies which have compared MR-CT CTV volumes with MR-only volumes for the same patient and found significant reductions when using MR-only.^3^^,^^7^ This suggests that MR-only is required to have the full benefit of MR for prostate CTV delineation. There was no statistically significant difference in OAR volumes, implying no systematic difference between the different pathways.

The dose calculation quality assurance results showed good agreement with previously reported data, with mean dose differences agreeing within 0.2%^13^ and only 3/96 patients outside the tolerance levels. Further investigation for these three patients showed clear anatomical changes in the patients which explained the out of tolerance results. There is a systematic dose difference between CBCT and CT when using the bulk density over-ride for prostate plans of −0.7%.^13^ Therefore, the majority of the mean dose difference of −0.9% is due to the CBCT calculation uncertainties, with a residual systematic dose difference from sCT of −0.2%. Therefore, the doses calculated by the MR-only pathway can be compared directly to those calculated on MR-CT and CT-only pathways with a negligible dose uncertainty. There was also no clinically significant difference in PTV doses between the three pathways, suggesting that PTV coverage was maintained equally between the pathways.

There were statistically significant dose differences to some of the OARs, namely the penile bulb mean dose and the high doses to the rectum (D$2\;{\text{cm}}^{3}$ and V60 Gy). MR is reported to have an improved visualization of the prostate apex and anterior rectal wall.^20^ A delineator contouring a target volume with an unclear boundary will tend to err on the side of being too big. Therefore, the improved MR visualization of the prostate apex and anterior rectal wall would give delineators more confidence in detecting the boundary, and so reducing the CTV size in these directions compared to MR-CT and CT-only. Given the steep dose fall-off outside the PTV achievable by VMAT techniques, this can result in significant dose sparing of the OAR. For the penile bulb there appears to be a trend with the CT-only pathway having the largest doses (median 12.0 Gy), the MR-CT fusion pathway intermediate doses (median 9.1 Gy), and the MR-only pathway the lowest (median 5.9 Gy, see Figure 4). This suggests that MR-CT fusion does improve delineators’ accuracy in detecting the prostate apex, but not as much as the MR-only pathway. Again, implying MR-only is necessary to get the full benefit of MR for delineation. The difference in the penile bulb mean dose is large (6.1 Gy), with the median MR-only dose being under half the median CT-only dose. There was also a corresponding increase in the percentage of patients within the dose constraint, suggesting these differences in doses would reduce the risk of erectile dysfunction for these patients.^15^ Together this suggests this is a clinically significant difference, especially as erectile dysfunction is the most commonly reported side-effect of prostate radiotherapy.^21^

The other statistically significant differences were in the higher doses to the rectum (D$2\;{\text{cm}}^{3}$ and V60 Gy), with the MR-only patients having the lowest doses. The difference in median D$2\;{\text{cm}}^{3}$ was only 0.9 Gy between MR-only and CT-only, although this corresponds to an increase in the odds of developing gastrointestinal toxicity grade ≥2 by 1.17.^16^ There were also larger changes in the proportion of patients within the V60 Gy<0.01% constraint,^17^ increasing from 35.1% (CT-only) to 56.2% (MR-only). These higher rectum doses are associated with increased risk of rectal bleeding,^17^ again suggesting these differences, though small, are likely to clinically as well as statistically significant. The variation in penile bulb and rectal doses between patients within each pathway were larger than the differences between pathways. Nonetheless, the statistically significant changes suggest that those differences are systematic between the pathways, and so on the population level implementing MR-only would lead to a small, but systematic reduction in penile bulb mean dose and high doses to rectum.

There were no statistically significant differences in the bladder D$2\;{\text{cm}}^{3}$ or the lower doses to the rectum (V50 Gy and V40 Gy) between the different pathways, although in all cases the median value was lowest for the MR-only pathway. The volumes of the rectum receiving lower doses will likely be much less impacted by the precise delineation of the prostate-rectum boundary, explaining why the differences were not statistically significant. The difference in visualization of the prostate-bladder boundary is also much less between MR and CT,^20^ hence, the lack of difference in the D$2\;{\text{cm}}^{3}$.

To the best of the authors’ knowledge, no study has evaluated the dose differences in clinical practice for prostate MR-only radiotherapy. Bird et al independently contoured target and OAR contours on CT and MR images of the same patient and compared dose differences between plans optimized on each image set.^22^ They reported statistically significant reductions in gross tumour volume of 47% (anus) and 57% (rectum), with corresponding statistically significant OAR dose reduction in bladder, uterus, penile blb and genitalia. This result accords with the results in this study, although demonstrating significantly larger volume reductions than found here (13%). This is likely due to Bird et al comparing MR-only and CT-only volumes and doses directly in the same patient, and so avoiding the large inter-patient variation, rather than comparing cohorts of patients treated on each pathway.

A limitation of this study is the fact that only planned doses have been compared. The dose constraints selected were those with dose-effect relationships demonstrated in the literature and proportions of patients within dose constraints derived from patient outcomes were used where possible to ensure the comparisons were on clinically relevant parameters. Nonetheless, there are significant uncertainties in those dose-effect relationships and so the predicted differences in patient outcomes for erectile dysfunction and rectal bleeding would need to be evaluated using patient outcome data. Another limitation of this study is the assumption that the OAR contour on CT for the CT and MR-CT patients is equivalent to the OAR contour on MR for the MR-only patients. Although this was necessary given the data available, the poorer soft-tissue contrast of CT is likely to worsen the contouring of OARs as well as targets. It is difficult to predict what impact this might have on doses to OARs, but it does add additional uncertainties in the OAR doses in CT-only and MR-CT pathways. A third limitation is the impact of inter-observer variability from the multiple delineators involved. This will be unlikely to bias the results as the same group of delineators performed all the delineations in each pathway.

In conclusion, patients treated with a MR-only prostate radiotherapy have statistically significant reduction in median CTV volume of $5\;{\text{cm}}^{3}$ compared to CT-only patients (P=.004). This resulted in statistically significant reductions in median penile bulb Dmean from 12.0 Gy (CT-only) to 5.9 Gy (MR-only, P<.001) and in rectum D$2\;{\text{cm}}^{3}$ from 57.4 Gy to 56.5 Gy (P<.001). These are likely to be clinically significant differences as the proportion of patients within constraints for penile bulb Dmean were 79.0% (CT-only) and 95.8% (MR-only) and for the rectum V60 Gy were 35.1% (CT-only) and 56.2% (MR-only). This suggests that MR-only prostate radiotherapy may result in reduced erectile dysfunction and rectal bleeding, although this needs to be validated using patient outcomes in a prospective study.

## Data Availability

Research data are stored in an institutional repository and will be shared upon request to the corresponding author.
